# Identification of Osteosarcopenia by High-Resolution Peripheral Quantitative Computed Tomography

**DOI:** 10.3390/jpm14090935

**Published:** 2024-09-02

**Authors:** Keith Yu-Kin Cheng, Simon Kwoon-Ho Chow, Vivian Wing-Yin Hung, Zoey Tsz-Lok Tsang, Benjamin Hon-Kei Yip, Ronald Man Yeung Wong, Ning Zhang, Ling Qin, Sheung-Wai Law, Wing-Hoi Cheung

**Affiliations:** 1 Bone Quality and Health Centre, Department of Orthopaedics and Traumatology, The Chinese University of Hong Kong, Shatin, Hong Kong SAR, China; keithykcheng@link.cuhk.edu.hk (K.Y.-K.C.);; 2 JC School of Public Health and Primary Care, The Chinese University of Hong Kong, Shatin, Hong Kong SAR, China; 3 Li Ka Shing Institute of Health Sciences, The Chinese University of Hong Kong, Shatin, Hong Kong SAR, China

**Keywords:** HR-pQCT, osteopenia, osteoporosis, osteosarcopenia, sarcopenia

## Abstract

Osteosarcopenia is a prevalent geriatric disease with a significantly increased risk of adverse outcomes than osteoporosis or sarcopenia alone. Identification of older adults with osteosarcopenia using High-Resolution Peripheral Quantitative Computed Tomography (HR-pQCT) could allow better clinical decision making. This study aimed to explore the feasibility of HR-pQCT to differentiate osteoporosis, sarcopenia, and osteosarcopenia in older adults, with a primary outcome to derive a model to distinguish older adults with osteosarcopenia from those with low bone mineral density only, and to examine important HR-pQCT parameters associated with osteosarcopenia. This was a cross-sectional study involving 628 community-dwelling Chinese adults aged ≥ 40. Subjects were assessed by dual energy X-ray absorptiometry (DXA) for osteopenia/osteoporosis and sarcopenia using the Asian Working Group for Sarcopenia definition; then grouped into healthy, osteopenia/osteoporosis, sarcopenia, and osteosarcopenia groups. A series of regression analyses and other statistical tests were performed to derive the model. HR-pQCT showed the ability to discriminate older adults with osteosarcopenia from those with osteopenia/osteoporosis only. Cross-validation of our derived model correctly classified 77.0% of the cases with good diagnostic power and showed a sensitivity of 76.0% and specificity of 77.6% (Youden index = 0.54; AUC = 0.79, *p* < 0.001). Analysis showed trabecular volumetric bone density and cortical periosteal perimeter were important and sensitive parameters in discriminating osteosarcopenia from osteopenia/osteoporosis subjects. These findings demonstrated that HR-pQCT is a viable and effective screening method for differentiating osteosarcopenia from low bone mineral density alone without the need to carry out multiple assessments for osteosarcopenia, especially for case-finding purposes. This could facilitate the decision of a follow-up and the management of these frail older adults to ensure they receive timely therapeutic interventions to minimise the associated risks.

## 1. Introduction

Osteoporosis and sarcopenia are highly prevalent geriatric diseases [1] which are associated with increased risk of adverse outcomes such as fractures and impaired quality of life [2]. Osteoporosis is defined as low bone mineral density and deteriorated bone microarchitecture, while sarcopenia is characterized by a progressive and generalized loss of skeletal muscle mass and strength. Osteosarcopenia is a newly emerging term to characterise individuals with both sarcopenia and osteopenia/osteoporosis, which has been found to be associated with a further increased risk of adverse events compared to those with only one of the diseases [3,4,5]. These age-associated geriatric conditions have been exacerbated by the recent global pandemic of coronavirus disease since 2019, as elderly people are becoming more susceptible to both osteoporosis and sarcopenia due to social distancing measures leading to a more sedentary lifestyle [6]. Currently, the diagnosis of osteosarcopenia requires many separate clinical assessments including muscle functional and performance tests, multiple bone density scans, and a whole-body composition assessment by dual energy X-ray absorptiometry (DXA), all of which impede the ease of identification of osteosarcopenia in busy clinical settings. Due to the severe implications of osteosarcopenia in older adults and time-consuming series of diagnostic tests currently required to identify osteosarcopenia, a convenient way to distinguish older adults with osteosarcopenia from those with osteopenia/osteoporosis only will be very beneficial for the early detection of osteosarcopenia. Once the likelihood of osteosarcopenia is determined by a simple and convenient test/scan, further confirmation of osteosarcopenia can be considered and management plans for these frail individuals can be recommended to minimise the risk of future adverse outcomes and the subsequent socioeconomic burdens.

DXA is the gold standard for clinically diagnosing osteoporosis and for defining low skeletal muscle mass as part of the sarcopenia diagnosis. Despite the benefits of DXA, technical limitations such as the inability to differentiate cortical and trabecular bone and its two-dimensional measurements have been scrutinized in recent years. These limitations are evident in studies that have shown around 50–80% of those experiencing fractures did not have a low areal bone mineral density (BMD) by DXA in adults aged ≥ 65 [7], which potentially compromised the identification of individuals who needed appropriate and timely treatments for poor bone quality.

High-Resolution Peripheral Quantitative Computed Tomography (HR-pQCT) is a three-dimensional imaging modality that non-invasively measures bone geometry, volumetric bone density, and bone microarchitecture. Unlike two-dimensional imaging methods, HR-pQCT provides more detailed information with higher resolution, all while having a short scan duration with minimal radiation exposure. HR-pQCT can overcome many limitations of DXA and has been found to have the ability to predict the risk of fragility fractures more accurately than DXA [8,9]. By performing routine scans, it has the potential to simultaneously assess the risk and likelihood of conditions such as osteoporosis, sarcopenia, and/or osteosarcopenia. Its ability to differentiate individuals with severe microstructural deterioration could be pivotal for follow-up care in geriatrics to improve risk management. This could be advantageous, since traditionally, diagnosing these conditions requires multiple clinical assessments and imaging scans, which could be time-consuming, and thus limit the convenience of case finding. Moreover, studies have shown that different physical activities can positively affect bone mass and microarchitecture [10,11], therefore it is highly probable that structural information generated by 3D imaging technology could be more widely used as a diagnostic tool in the future to identify morphologies associated with diseases and to facilitate clinical management [12].

Therefore, the objectives of this study were to explore the feasibility of whether HR-pQCT can differentiate osteoporosis, sarcopenia, and osteosarcopenia in older adults, to derive a validated model to distinguish osteopenia/osteoporosis from osteosarcopenia, and to examine the association between HR-pQCT parameters and osteosarcopenia risk in older adults with low bone mineral density.

## 2. Materials and Methods

### 2.1. Study Design

**Figure 1:**
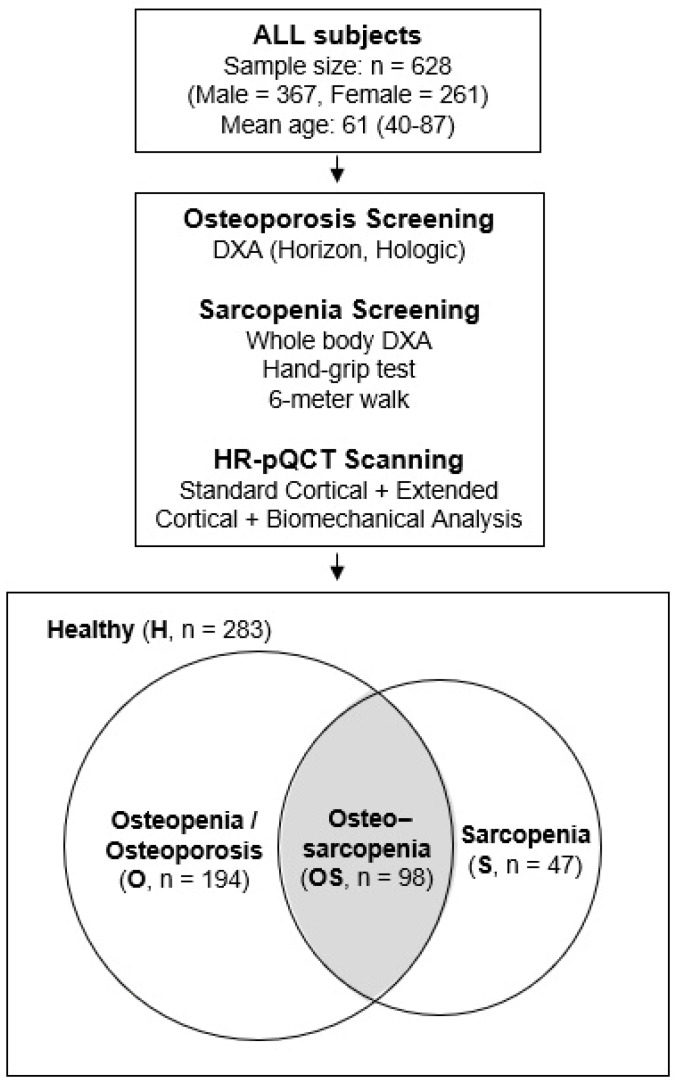
Study design summary and statistics.

This is a retrospective, cross-sectional study which included 628 community-dwelling Chinese adults in Hong Kong, aged 40 and above, who received bone and muscle assessments at the Prince of Wales Hospital. Subject data were extracted from a published database and the clinical approach to data collection has been described in our previous publication [13]. In brief, subjects were able to walk independently, had no history of fragility fractures, and had not received osteoporosis treatment recently. Those with conditions or medications that could affect bone and muscle metabolism were excluded to control for confounding variables. Subjects were assessed by DXA to determine their bone mineral density defined by WHO and screened for sarcopenia based on the Asian Working Group for Sarcopenia definition in 2019 (AWGS 2019) [14]. Additional HR-pQCT data were also available to examine microarchitecture and biomechanical parameters for further analysis on bone quality (Figure 1). All procedures and assessments were conducted in the Prince of Wales Hospital (Bone Quality and Health Centre, Department of Orthopaedics and Traumatology, The Chinese University of Hong Kong). Ethics were approved by the Joint Chinese University of Hong Kong–New Territories East Cluster Clinical Research Ethics Committee (CRE.2014.310). All participants provided written informed consent, and all research were performed in accordance with relevant guidelines and regulations and the Declaration of Helsinki. Subjects were divided into 4 groups for analysis based on their status, namely Healthy (H, for those without any of the disease states in question), Osteopenia/Osteoporosis only (O, for those with low BMD with T-score < −1), Sarcopenia only (S), and Osteosarcopenia (OS) groups. Subjects eligible for both osteopenia/osteoporosis and sarcopenia groups were only included in the OS group for analysis.

### 2.2. Diagnosis of Osteoporosis and Grouping

**Figure 2:**
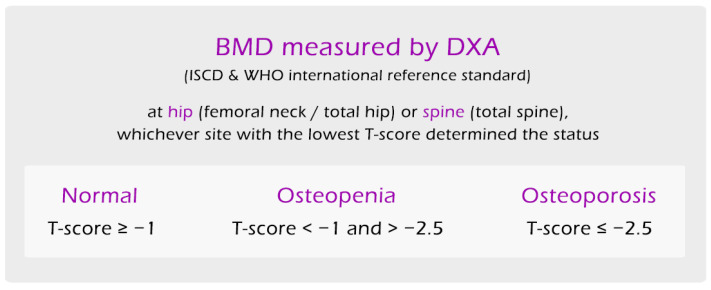
Diagnostic criteria for low bone mineral density (osteopenia/osteoporosis) used in this study.

BMD was assessed at lumbar spine and hip using DXA (Horizon, Hologic, Marlborough, MA, USA) by independent certified technicians. Osteoporosis and osteopenia, or low bone mineral density, were defined by BMD T-scores based on the WHO international reference standard, with the hip (femoral neck or total hip) or spine (total spine) T-score < −1 and >−2.5 being osteopenia and T-score ≤ −2.5 being osteoporosis, and whichever site had the lowest T-score determined the status (Figure 2). Calibration of the DXA machine was done using bone phantom every day, which gave an acceptable precision error of 1.31% for total hip and 0.72% for spine [15]. Since BMD has been reported to be lost at 0.3–0.5% per year for adults over age 30 [16], subjects between 40–49 years old with T-scores < −1 were grouped together with other subjects with low BMD in the Osteopenia/Osteoporosis group for the purpose of this analysis. Individuals younger than 50 or premenopausal women are not typically screened for bone density based on current guidelines; however, this younger demographic was included to provide insight into the bone density and microarchitectural characteristics of a healthier subgroup to better identify at risk individuals.

### 2.3. Diagnosis of Sarcopenia

**Figure 3:**
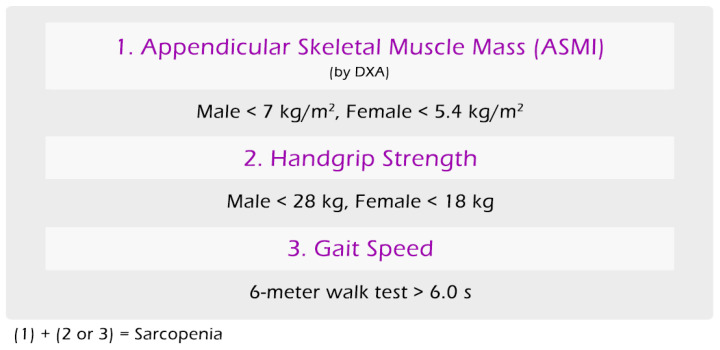
Diagnostic criteria for sarcopenia used in this study.

Whole body composition assessment by DXA were used to assess appendicular skeletal muscle mass by segmented measurement. Sarcopenia diagnosis was based on the AWGS 2019 sarcopenia definition, in which the combination of low appendicular muscle mass (by DXA: male at <7 kg/m^2^, female at <5.4 kg/m^2^) and either low handgrip strength (by dynamometer: male at <28 kg, female at <18 kg) or low gait speed (by 6 m walk test > 6.0 s) was considered to have sarcopenia (Figure 3).

### 2.4. HR-pQCT Measurement

The measurement protocol was described in greater detail in our previous publication [13] and is described in brief below. For technical details and algorithms used in the calculations, please refer to Scanco Medical user manuals and existing literatures [17,18]. To summarize, distal radius and distal tibia of the non-dominant side of the subjects were scanned by HR-pQCT (XtremeCT, Scanco Medical AG, Brüttisellen, Switzerland) using the standard clinical in vivo scanning protocol (60 kVp, 900 μA, 100 ms integration time, isotropic voxel size 82 mm) [13]. Scans were repeated if motion was detected. All images were graded by a single operator and only good quality images with no motion artifact or only minor motion artifact (grade 1 and 2) were used for this study, based on the grading system proposed by Pialat and colleagues [19]. Good intrarater reliability was concluded from an examination of 120 images which showed a linear weighted kappa of 0.855.

The standard analysis, extended cortical analysis [17], and biomechanical properties estimated by micro-finite element (µFE) analysis were investigated, with 134 variables generated per subject for radius and tibia in total. The data were separated into cortical and trabecular components using an automated cortical compartment segmentation technique [20]. The contour was checked and corrected by a trained operator. Total, cortical, and trabecular volumetric BMD (vBMD) in mg hydroxyapatite (HA)/cm^3^ and area in mm^2^ were then calculated. The centre points of trabeculae or 3D ridges were identified and the spacing between them was assessed by the distance-transformation method. Trabecular number (mm^−1^) was defined as the inverse mean spacing of the 3D ridges. Trabecular thickness and separation were derived analogous to standard histomorphometry methods. Cortical thickness (mm) was derived from a direct 3D calculation of endosteal periosteal distance, performed on composite segmentations of the mineralized cortex, disregarding intracortical pore surfaces in these calculations. A volumetric index of intracortical porosity (Ct.Po, %) was calculated based on the cortical pore volume and the mineralized cortical bone volume, with the Equation shown below and details are further described in references [13,21].

$$Ct.Po=\frac{Ct.PoV}{Ct.PoV+Ct.BV}$$

Ct.PoV = segmented pore volume
Ct.BV = mineralized cortical bone volume

All µFE analyses were performed using the FE-solver included in the built-in Image Processing Language software IPL-FE v1.15 by Scanco Medical. A special peeling algorithm specifying a minimum cortical thickness of 6 voxels was used to identify cortical and trabecular bone tissue. µFE analyses were performed by converting the binary image data to a mesh of isotropic brick elements. For all elements, a Poisson’s ratio of 0.3 was specified. Elements representing cortical and trabecular bone were both assigned a Young’s modulus of 10 GPa. A uniaxial compression test with a 1000 N load applied was performed with an applied strain of 1%. Whole-bone stiffness (kN/mm) was calculated. Estimate failure load (N) was calculated based on the assumption that bone failure occurred if >2% of the elements were strained beyond 0.7% strain.

### 2.5. Statistical Analyses

All statistical analyses were computed using SPSS Version 26 (IBM Corp., Armonk, NY, USA). Geometric, volumetric, density and structural parameters from HR-pQCT standard analysis, extended cortical analysis and biomechanical analysis at the radius and the tibia were examined. Spearman’s rank test was performed to measure associations between all HR-pQCT independent variables to test their relationships. Variance inflation factor (VIF) analysis was used to assess how much each independent variable was inflated by the presence of other variables, known as multicollinearity, and VIF > 3 were eliminated to form a smaller selected pool of parameters for regression analysis.

A series of logistic regressions were performed to test the ability of the selected HR-pQCT parameters to classify subjects in different groups using one versus one and one versus rest multi-class classification methods. Subjects in the healthy group were considered the negative state in the regression analyses. Age was adjusted as a covariate in all regression models.

In order to discriminate and classify subjects in the O and OS groups, binary logistic regressions were deployed to analyse the selected HR-pQCT parameters to form a model to predict the probability of the two outcomes. Subjects were split into independent two-thirds as the learning set for regression analysis and one-third for cross-validation to test the derived regression model. Backwards stepwise method was used with the probability of 0.05 and 0.10 for the stepwise entry and removal criteria respectively. Independent samples T-tests were used to find significant differences in HR-pQCT parameters between O and OS groups. Adjusted odds ratio for certain HR-pQCT parameters found to have high association in regression analysis were reported to characterise bone characteristics of OS subjects in comparison to those with O only. Classification tables generated by regression analyses were used to report sensitivity and specificity, and the associated Youden index (Youden’s index (J) = sensitivity% + specificity% − 100) were calculated to assess the model’s diagnostic power. Receiver operating characteristic (ROC) curves were used to determine the optimal probability cut-off for case classifications for each of the regression models. Sub-analysis analysed each sex (male and female) separately using the same statistical approach; these data are included in the Supplementary Materials. *p*-value < 0.05 was considered significant in all analyses.

## 3. Results

Among 628 Chinese adults, 58% were male and 42% were female. The mean age was 60 (40–84, SD: 12) for male and 63 (40–87, SD: 11) for female. In males and females, respectively, the prevalence of osteopenia/osteoporosis was 35.7% and 60.2% (in subjects age ≥ 50), sarcopenia was 19.1% and 28.3%, and osteosarcopenia was 9.6% and 23.2%. Male with sarcopenia was found to be 2.1 times more likely (OR = 2.1; 95%CI: 1.23, 3.58; *p* < 0.01) to have low BMD (osteopenia/osteoporosis), compared to female with sarcopenia at 4.3 times more likely (OR = 4.3, 95%CI: 2.18, 8.28; *p* < 0.001). Total hip and spine BMD T-scores were significantly different among all groups (H/O/S/OS) for male and female, except at the spine between O and OS groups for both male (*p* = 1.0) and female (*p* = 0.197), and between healthy and sarcopenia groups at the spine for male only (*p* = 1.0) (Table 1) where no significant differences were found using DXA.

### 3.1. Discriminating Subjects with Different Health Status Using HR-pQCT

A total of 26 geometric, volumetric, density, and structural parameters from HR-pQCT were selected for this regression analysis after accounting for collinearity (Table 2). A series of binary logistic regressions were computed using these HR-pQCT parameters to assess the ability to discriminate subjects with different disease status from healthy control H group. H and O group comparison (*n* = 397) found a sensitivity of 89% and specificity of 75.8% in identifying subjects with O, and AUC from regression was 0.90 (*p* < 0.001). H and S group comparison (*n* = 293) yielded similar results, with 87.8% sensitivity and 75.4% specificity with AUC = 0.88 (*p* < 0.001). H and OS group comparison (*n* = 325) had 90.4% sensitivity and 90.9% specificity, with AUC = 0.97 (*p* < 0.001) (Table 3A).

Further regression analysis comparing subjects with O to the rest of the subjects without O (H + S) found a sensitivity of 86.2% and specificity of 72.7% for O subjects with AUC = 0.88 (*p* < 0.001). Another comparison between S and the rest of the subjects (H + O) found a sensitivity of 90.2% and specificity of 61.5% for S subjects with AUC = 0.822 (*p* < 0.001) (Table 3B).

### 3.2. Differences between Osteoporotic (O) and Osteosarcopenic (OS) Subjects by HR-pQCT

For the HR-pQCT radius and tibia scans, each scan generated a total of 134 parameters in the standard analysis, extended cortical analysis and biomechanical analysis. In subjects aged ≥ 65 years, 29 (21.6%) and 57 (42.5%) HR-pQCT parameters for male and female respectively were found to be significantly different between O and OS groups (*p* < 0.05). As the extended cortical analysis uses a dual-threshold segmentation technique to provide more robust extraction of the cortical and trabecular compartments [17], more parameters from the extended cortical analysis were found significantly different between O and OS groups than parameters from standard analysis.

Large significant differences were found in the moment of inertia (MOI) parameters for both radius and tibia in both sexes, showing significantly worse bone strength and stiffness in OS group compared to O. A few notable parameters were found to have high associations with OS and showed significant differences. Cortical periosteal perimeter at both sites were significantly lower in OS group for males (radius: *p* < 0.05; tibia: *p* < 0.01) and female (radius: *p* < 0.001; tibia: *p* < 0.001). A significantly lower total bone volume in tibia (*p* < 0.001), standard deviation of the cortical von Mises stress in tibia (*p* < 0.05), total cortical bone volume in radius (*p* < 0.05), and estimated failure load in tibia (*p* < 0.001) were found for females in OS compared to O. A significantly larger cortical pore diameter (*p* < 0.05) was found in tibia for females in OS compared to O.

#### Discriminating OS from O Subjects in Mixed Sex Analysis with Validation

Logistic regression analysis was conducted using a smaller selection of parameters after accounting for collinearity as described in the methods. The aim was to evaluate the capability of HR-pQCT in identifying osteosarcopenia among subjects with osteopenia/osteoporosis (*n* = 292). The data were separated into an independent two-thirds as the regression group for training and the remaining one-third as the validation group for cross-validation, while ensuring equal distribution of age and sex. A logit function was derived from regression analysis (Table 4) using the regression group (n = 218) to predict the probability of also having sarcopenia (osteosarcopenia) in an individual with osteopenia/osteoporosis, as follows:

$$\begin{array}{ll} Logit\left(P\right)= & 18.273+13.260\left(ExtT\;Ct.Po\right)-0.179\left(StdT\;Ct.Pm\right)\\ & -0.035\left(StdT\;Dtrab\right)+8.612(BmT(Tb.F/TF)Prox)\\ & +0.061(Age)-2.542(Sex;M=1,F=2) \end{array}$$

ExtT = Extended cortical analysis of the tibia
StdT = Standard analysis of the tibia
BmT = Biomechanical analysis of the tibia
Ct.Po = Cortical porosity
Ct.Pm = Cortical periosteal perimeter
Dtrab = Trabecular bone density
(Tb.F/TF)Prox = Ratio between the load supported by trabecular bone and the load supported by the whole bone at the proximal end

The overall model was statistically significant compared to the null model (χ^2^(6) = 61.6, *p* < 0.005), explained 34.2% (Nagelkerke *R*^2^) of the variance in osteosarcopenia, and correctly classified 71.6% of cases. The Hosmer–Lemeshow test had a result of χ^2^(8) = 3.39, *p* = 0.908 showing good fit between data and model.

With the standardization into Z-scores, for each standard deviation (SD) unit increase in (Tb.F/TF)Prox in tibia, the odds of having osteosarcopenia in those with osteopenia/osteoporosis increased by 92% (1.92, 95% CI: 1.15–3.22). Other notable parameters included that cortical porosity increased odds by 44% (OR: 1.44, 95% CI: 0.96–2.15), cortical periosteal perimeter decreased odds by 83% (OR: 0.17, 95%CI: 0.086–0.341) and trabecular bone density decreased odds by 63% (OR: 0.37, 95%CI: 0.211–0.633) for every one-unit increase in the standard deviation (SD). One year increase in age increased the odds of OS by 6.3% (OR: 1.063, 95%CI: 1.018–1.109). Female with low BMD was found to be 12.7 times more likely in odds to have OS compared to male (OR: 12.7, 95%CI: 3.331–48.421) (Table 4). ROC curve for the regression group showed an area under curve (AUC) of 0.80 (*p* < 0.001) and the optimal probability cut-off was determined at 0.354, above which was determined as having osteosarcopenia. In the validation group (*n* = 74), the remaining one-third, when tested on the regression model derived above, showed good diagnostic ability with AUC = 0.79 (*p* < 0.001). The main predictor, trabecular bone density in the standard analysis of tibia (β = −1.01, OR = 0.37, *p* < 0.005), has an effect size of 0.4. The power of the analysis was found to be 0.881 as calculated by G*Power 3.1 with alpha = 0.05. (df = 278, Critical t = 1.97, noncentrality parameter = 3.15).

Overall, the sensitivity and specificity for the regression group was 75.3% and 75.2% respectively, while the validation group using the regression model correctly classified 77.0% of the cases and showed a sensitivity of 76.0% and specificity of 77.6% (Table 5). In the cross-validation, the Youden index was found to be 0.54, which is greater than 0.50, indicating good diagnostic power and accuracy for this model in discriminating osteopenia/osteoporosis and osteosarcopenia.

Sex specific analyses for male only (AUC = 0.87, *p* < 0.001, Youden Index = 0.62) and female only (AUC = 0.83, *p* < 0.001, Youden Index = 0.53) using a similar statistical approach are available in the Supplementary Materials.

## 4. Discussion

The relationship between bone and muscle has been increasingly studied to examine associations of certain characteristics to particular diseases or conditions [9,22,23,24]. The musculoskeletal unit has been found to deteriorate together with disuse and aging, and the association between osteoporosis and sarcopenia has been well reported in the past that the reduction of muscle mass and strength are significantly associated with decreased BMD, bone mass, and deteriorated bone microarchitecture [25,26]. As early as in embryonic development, bone morphology and microarchitecture are shaped by forces exerted by muscle to form mechanically functional bones [27]. Throughout life, skeletal muscle contractions exert forces that cause mechanical and elastic deformations of bone leading to the appropriate bone growth and remodelling, known as biomechanical coupling of the mechanostat theory [28]. This gives rise to the potential to assess muscle-related conditions based on the effect of muscle disuse and loss to aging evident in bone microarchitecture. In this study, HR-pQCT bone microarchitecture parameters were shown to have the ability to discriminate osteopenia/osteoporosis (O), sarcopenia (S) and osteosarcopenia (OS) in Chinese older adults, with 90.8% accuracy in discriminating OS from healthy subjects (Table 4). More importantly, the potential ability of HR-pQCT to identify characteristics unique to the contribution of sarcopenia in osteosarcopenia can be useful in providing further diagnostics information. To further this finding, our derived regression model in our mixed sex analysis performed well in discriminating OS from O and correctly classified 77.0% of the cases in cross-validation with a high sensitivity of 76.0% and specificity of 77.6% showing good diagnostic power and accuracy for OS (Youden index = 0.54 and AUC = 0.8, *p* < 0.001). Among all parameters with significant associations with OS in regression, trabecular volumetric bone density was the only parameter found significant in all mixed sex, male and female analyses consistently, showing trabecular volumetric bone density as one of the more important parameters in discriminating older adults with osteosarcopenia from those with low bone density only. To our knowledge, this is one of the first studies to analyse bone microarchitecture parameters of HR-pQCT comparing sarcopenia and osteosarcopenia.

Trabecular volumetric bone density (*p* < 0.01) and cortical periosteal perimeter (*p* < 0.01) were two parameters in mixed sex regression analysis found to have the highest association with OS compared to O, and the reduction in both greatly increased the risk of OS. Both of these have been reported to decrease with age, especially when associated with osteoporosis [8,29]. This loss of volume and density of the more metabolically active trabecular bone has been observed in early osteoporosis of postmenopausal women [30], and our study found this deterioration was sensitive in the discrimination of OS in older adults with O. Between these two parameters, a significantly lower cortical periosteal perimeter was found only in females aged ≥ 65 with OS compared to O (*p* < 0.01).

The sex specific analysis (refer to Supplementary Materials) also found trabecular volumetric bone density to have significant association with OS compared to O (*p* < 0.05 for both male and female). In addition, bone volume and cortical thickness were other parameters found significant in both male and female specific analyses. Aside from the loss in bone volume and density as discussed earlier, cortical thickness reduction as a result of cortical trabecularization is commonly found in altered bone geometry in osteoporosis, especially in individuals with less physical activities [31,32]. Reduction in cortical thickness in the radius for male and in the tibia for female was found to be a sensitive measure for the prediction of OS. Furthermore, in the male radius, cortical porosity and trabecular separation were found to be significantly associated with OS, and both of these parameters have been shown to increase with aging [30,33,34,35]. Cortical porosity is represented by the average fraction of void volumes within the cortical bone volume, which is measured exclusively by HR-pQCT and can offer unique assessment of cortical bone strength that DXA cannot measure [8]. Lastly, in the male tibia, cortical bone polar moment of inertia (pMOI), which is a microstructural estimation of resistance to torsion that reflects the bone’s biomechanical ability to withstand twisting forces [36], was found to be significantly associated with OS, and a significant difference was found between male OS and O subjects showing association between sarcopenia and the deterioration of tibia bone strength in older osteopenic/osteoporotic men. Studies have found that certain interventions, such as aerobic exercise training, can significantly improve trabecular bone microarchitecture [10,11]; hence, 3D imaging techniques like HR-pQCT may pave a way to targeted therapy for subjects with specific microarchitecture deteriorations at an elevated risk of adverse outcomes such as fractures.

DXA is the gold standard for osteopenia/osteoporosis and sarcopenia diagnosis; however, it requires scans at the spine and the hip to determine osteoporosis and another whole-body scan in addition to a series of muscle function and performance tests to diagnose sarcopenia. Our study found that DXA was unable to identify significant differences in spine BMD in osteosarcopenia subjects compared to those with osteopenia/osteoporosis only, potentially making it an insensitive measure of the further deteriorated bone quality in osteosarcopenia. These standard scans from HR-pQCT have promising potential as an alternative for case-finding, screening, or risk assessment for various bone and muscle related diseases, with up to 90% sensitivity and specificity found in our study (Table 3A) [8,37]. Instead of exposing the entire body to ionizing radiation [38], quick scans using HR-pQCT alone have been demonstrated to have good accuracy and good diagnostic power based on our findings in differentiating among osteoporosis, sarcopenia, and osteosarcopenia by scanning the radius and/or tibia. This approach also saves time by reducing the number of additional clinical assessments that must be completed in the case-finding process. This efficient identification of older adults with low BMD at risk of osteosarcopenia, compounded with severe bone microstructural deterioration and compromised muscle performance could guide therapeutic interventions for a timelier follow-up and the management of these frail older adults.

This study has its limitations. A larger sample size is needed for cross-validation on sex specific analysis of O and OS subjects to validate the sex specific regression models in the sub-analysis section. Secondly, despite high diagnostic power found in the mixed sex cross-validated analysis to discriminate osteosarcopenia from those with osteopenia/osteoporosis alone, the specificity was 77.6%, which may lead to some false positive cases. However, for the purpose of risk assessment and probable case-finding, false positive cases of osteosarcopenia will have minimal adverse health effects while beneficially raising awareness for a healthier lifestyle. Further analysis of the muscle and soft tissue at the existing scanned areas could improve the accuracy significantly. Follow-up assessments to check for sarcopenia can verify without additional discomfort or significant costs using community screening methods for high risk individuals [39]. Currently, HR-pQCT is an expensive device, and a significant cost reduction by large-scale manufacturing may be needed to improve accessibility in clinical practice. Before fully utilizing HR-pQCT for medical diagnosis, further research is needed to determine cut-off values that correspond to adverse outcomes or poor performance defined by the disease. Regardless, the results of this study offer compelling proof that HR-pQCT can be used to identify distinct bone microarchitectural changes in diseases such as osteosarcopenia.

In conclusion, HR-pQCT measurement was shown to have the potential for accurate identification of osteoporosis, sarcopenia, and osteosarcopenia in older adults. Our cross-validated regression model confirmed that HR-pQCT can discriminate osteosarcopenia subjects from those with osteopenia/osteoporosis only with high accuracy. High associations were found in trabecular volumetric bone density and cortical periosteal perimeter of the tibia with osteosarcopenia, and the reduction in both could reflect the risk of osteosarcopenia. The utilization of this new generation imaging technology could enable the identification of a wider array of diseases in less scans and provide more comprehensive risk assessments.

## Figures and Tables

**Table 1:** Table showing BMD T-score characteristics by DXA for male and female subjects in each of the groups. No significant differences were found at the spine between Osteopenia/Osteoporosis group and Osteosarcopenia group for both male and female.

**Male**	**Healthy**			**Osteopenia/Osteoporosis**			**Sarcopenia**			**Osteosarcopenia**		
	*n*	Mean ± SD	Range	*n*	Mean ± SD	Range	*n*	Mean ± SD	Range	*n*	Mean ± SD	Range
T-score Total Spine	195	1.1 ± 1.4	(−1, 6.1)	93	−0.8 ± 1.1	(−2.8, 2.4)	34	1.4 ± 1.6	(−1, 5.8)	34	−1.2 ± 1.4	(−3.3, 4.1)
T-score Total Hip	195	0.7 ± 0.8	(−1, 4.2)	93	−0.4 ± 0.6	(−1.9, 1.4)	34	0.3 ± 0.7	(−1.2, 2)	34	−0.9 ± 0.6	(−2.4, −0.1)
**Female**	**Healthy**			**Osteopenia/Osteoporosis**			**Sarcopenia**			**Osteosarcopenia**		
	*n*	Mean ± SD	Range	*n*	Mean ± SD	Range	*n*	Mean ± SD	Range	*n*	Mean ± SD	Range
T-score Total Spine	88	0.9 ± 1.4	(−1, 7.4)	94	−1.6 ± 1	(−3.9, 1.4)	13	0 ± 0.8	(−0.9, 1.8)	59	−2 ± 1.1	(−4.9, 0.2)
T-score Total Hip	88	1.2 ± 0.9	(−0.8, 3.7)	94	−0.4 ± 0.8	(−2.4, 2.3)	13	0.4 ± 0.6	(−0.7, 1.7)	59	−1 ± 0.9	(−3.2, 1.1)

**Table 2:** List of selected parameters used in the regression analyses to discriminate subjects with different health status using HR-pQCT. Trab, trabecular; Ct, cortical; Ct.VM, Cortical von Mises stress; BMD, bone mineral density; Trab.VM, Trabecular von Mises stress; Meta/Inn, Ratio of meta to inner trabecular bone density.

	Radius	Tibia	Demographics
Extended cortical analysis	Ct. Pore Volume, mm^3^	Total volumetric BMD, mgHA/cm^3^	
	Structural Model Index	Ct. BMD, mgHA/cm^3^	
	Trab. Number, per mm	Ct. Bone volume, mm^3^	
	Trab. Thickness, mm	Ct. Pore Volume, mm^3^	
	Total section modulus relative to larger main axis of inertia, mm^3^	Ct. Pore Diameter, mm	
		Trab. Number, per mm	
		Trab. BMD, mgHA/cm^3^	
Standard analysis	Trab. Area, mm^2^	Trab. Area, mm^2^	
	Average Bone Density, mgHA/cm^3^	Trab. Thickness, mm	
	Compact Bone Density, mgHA/cm^3^		
	Meta/Inn		
Biomechanical analysis	Ratio between load supported by trabecular bone versus whole bone, at the distal end ((Trab.F/TF)dist)	Ct.VM	
	Average equivalent strain cortical bone	Standard deviation of Trab.VM	
	Ct.VM		
	Standard deviation of Ct.VM		
Others			Age
			Sex

**Table 3:** (**A**). Regression analysis showing one versus one multi-class classification for different disease states, with the “Healthy” group defined as negative state. (**B**). Regression analysis showing one versus rest multi-class classification for different disease states.

**(A)**	**Classification**				**Correct Classification**	**AUC (95% CI)**
	**Healthy**	**Osteoporosis/Osteopenia**	**Sarcopenia**	**Osteosarcopenia**		
Healthy	191	61	-	-	75.8%	-
Osteoporosis/Osteopenia	16	129	-	-	89.0%	-
					80.6%	0.90 (0.87–0.93)
Healthy	190	-	62	-	75.4%	-
Sarcopenia	5	-	36	-	87.8%	-
					77.1%	0.88 (0.83–0.93)
Healthy	229	-	-	23	90.9%	-
Osteosarcopenia	7	-	-	66	90.4%	-
					90.8%	0.97 (0.95–0.99)
**(B)**			**Classification**		**Correct Classification**	**AUC (95% CI)**
			**Negative**	**Positive**		
Osteoporosis/Osteopenia versus Rest	Negative (Healthy/Sarcopenia)		213	80	72.7%	-
	Positive (Osteoporosis/Osteopenia)		20	125	86.2%	-
					77.2%	0.88 (0.84–0.91)
Sarcopenia versus Rest	Negative (Healthy/Osteoporosis/Osteopenia)		244	153	61.5%	-
	Positive (Sarcopenia)		4	37	90.2%	-
					64.2%	0.82 (0.76–0.88)

**Table 4:** Binary logistic regression for the regression group for predicting osteosarcopenia (OS) in subjects with osteoporosis/osteopenia, showing predictor variables, β coefficients, and adjusted odds ratio (95%CI) for mixed sex analysis in O and OS groups (*n* = 218). OS was coded as the positive state in the analysis. OR, Odds ratio (predicts the change in odds for 1 standard deviation unit increase in the predictor variable); S.E., Standard error; ^Ŧ^ Male = 1 and Female = 2; Probability cut-off was determined at 0.354; * *p* < 0.05, ** *p* < 0.01.

		β	S.E.	Wald	β	S.E.	Odds Ratio	Sig.
					Z-Distribution Standardization			
Extended cortical analysis (tibia)	Cortical porosity, %	13.26	7.58	3.06	0.36	0.21	1.44	0.08
Standard analysis (tibia)	Cortical periosteal perimeter, mm	−0.18	0.04	25.36	−1.76	0.35	0.17	0.00 **
	Trabecular bone density, mgHA/cm^3^	−0.03	0.01	12.92	−1.01	0.28	0.37	0.00 **
Biomechanical analysis (tibia)	Ratio between load supported by trabecular bone versus whole bone, at the proximal end (Tb.F/TF)	8.61	3.46	6.18	0.65	0.26	1.92	0.01 *
	Age	0.06	0.02	7.62	-	-	1.06	0.01 **
	Sex ^Ŧ^	−2.54	0.68	13.86	-	-	12.70	0.00 **
	Constant	18.27	4.84	14.23	-	-	-	0.00 **

**Table 5:** Classification table for (**A**) regression group and (**B**) validation group in mixed sex regression analysis with O and OS; probability cut-off at 0.354.

**(A) Disease Status (Regression Group)**	**Classification**			
	**Osteoporosis/Osteopenia**	**Osteo-Sarcopenia**	**Total**	**Correct%**
Osteoporosis/Osteopenia	109	36	145	75.2%
Osteosarcopenia	18	55	73	75.3%
				75.2%
**(B) Disease Status (Validation Group)**	**Classification**			
	**Osteoporosis/Osteopenia**	**Osteo-Sarcopenia**	**Total**	**Correct%**
Osteoporosis/Osteopenia	38	11	49	77.6%
Osteosarcopenia	6	19	25	76.0%
				77.0%

## Data Availability

The dataset presented in this article is not readily available due to the data being part of an ongoing study. Requests to access the dataset should be directed to the corresponding author.

## Supplementary Materials

The following supporting information can be downloaded at: https://www.mdpi.com/article/10.3390/jpm14090935/s1.
