# Supplementary material for: Identification of Osteosarcopenia by High-Resolution Peripheral Quantitative Computed Tomography
Source: J Pers Med. 2024 Sep 2;14(9):935. doi: 10.3390/jpm14090935 (PMC11433288; doi:10.3390/jpm14090935)
Supplement: Supplementary file 1 [file jpm-14-00935-s001.zip › jpm-3139526-supplementary.pdf]

# 1 *Supplementary Materials*

## 1.1 *Discriminating OS from O Subjects in each sex: Analysis for Male over 65*

A logistic regression was performed to ascertain the effects of HR-pQCT parameters on the likelihood that male subjects aged ≥ 65 (n=49) with osteopenia/osteoporosis also had sarcopenia (osteosarcopenia). The model was statistically significant compared to null model ($\chi^2(13) = 22.8$, $p < 0.05$). The model explained 50.4% (Nagelkerke $R^2$) of the variance in osteosarcopenia male and correctly classified 81.6% cases.

Several parameters were found to be highly associated with OS in the final regression model with high Wald statistic. With Z-score standardization, each SD increase in polar moment of inertia (pMOI) of the tibia was found to have a protective effect in subjects with lower BMD by a 92.7% reduction in odds (OR: 0.073, 95%CI: 0.008 - 0.679). Among the predictor variables in the final model (Supplementary Table S1), a significant difference between O and OS group for male was found only in pMOI of tibia ($p < 0.01$).

Hosmer and Lemeshow test was $\chi^2(8) = 2.49$, $p = 0.962$ showing good fit between data and the model. ROC curve from the regression showed an AUC of 0.87 ($p < 0.001$) and the optimal probability cut-off was determined at 0.443. The sensitivity and specificity for this model was 78.9% and 83.3% respectively (Youden Index = 0.62).

## 1.2 *Discriminating OS from O Subjects in each sex: Analysis for Female over 65*

A logistic regression was performed to ascertain the effects of HR-pQCT parameters on the likelihood that female subjects aged ≥ 65 (n=71) with osteopenia/osteoporosis also had sarcopenia (osteosarcopenia). The model was statistically significant compared to null model ($\chi^2(3) = 23.0$, $p < 0.001$). The model explained 36.9% (Nagelkerke $R^2$) of the variance in osteosarcopenia female and correctly classified 76.1% cases.

Among the predictor variables in the final regression model, a significant difference between O and OS groups was found only in total bone volume in tibia for female ($p < 0.001$). With Z-score standardization, each SD increase in total bone volume in tibia (OR: 0.152, 95%CI: 0.058 - 0.400), cortical thickness in tibia (OR: 0.322. 95%CI: 0.133 - 0.780) and meta-trabecular bone density in tibia (OR: 0.481, 95%CI: 0.254 - 0.912) decreased the odds of OS in females with low BMD (Supplementary Table S2).

Hosmer and Lemeshow test was $\chi^2(8) = 8.82$, $p = 0.358$ showing good fit between data and the model. ROC curve from the regression showed an AUC of 0.83 ($p < 0.001$) and the optimal probability cut-off was determined at 0.480. The sensitivity and specificity for this model was 81.8% and 71.1% respectively (Youden Index = 0.53).

**Supplementary Table S1**. Binary logistic regression model for predicting osteosarcopenia in subjects with osteoporosis/osteopenia, showing predictor variables and the β coefficients for males aged ≥ 65 (n=49). Osteosarcopenia was coded as the positive state in the analysis. Probability cut-off was determined at 0.443; *p < 0.05, ** p < 0.01.

| | | β | S.E. | Wald | β | S.E. | Odds Ratio | Sig. |
|---|---|---|---|---|---|---|---|---|
| | | | | | **Z-distribution standardization** | | | |
| Extended cortical analysis (**radius**) | Apparent cortical thickness, mm | -14.37 | 6.88 | 4.37 | -3.24 | 1.55 | 0.04 | 0.04* |
| | Cortical porosity, % | -116.68 | 59.33 | 3.87 | -2.13 | 1.08 | 0.12 | 0.05* |
| | Cortical pore diameter, mm | 58.5 | 37.45 | 2.44 | 1.01 | 0.65 | 2.74 | 0.12 |
| Extended cortical analysis (**tibia**) | Cortical bone volume, $mm^3$ | 0.02 | 0.01 | 5.78 | 3.46 | 1.44 | 31.90 | 0.02* |
| | Cortical porosity, % | 63.66 | 32.99 | 3.72 | 1.63 | 0.85 | 5.10 | 0.05 |
| | SD of cortical pore diameter, mm | -90.37 | 45.71 | 3.91 | -1.30 | 0.66 | 0.27 | 0.05* |
| | Cortical bone polar moment of inertia, $mm^4$ | - | - | 5.29 | -2.62 | 1.14 | 0.07 | 0.02* |
| Standard analysis (**radius**) | Inner trabecular bone density, $mgHA/cm^3$ | 0.08 | 0.04 | 4.12 | 2.21 | 1.09 | 9.11 | 0.04* |
| | Trabecular separation, mm | 22.93 | 10.07 | 5.19 | 2.14 | 0.94 | 8.50 | 0.02* |
| Standard analysis (**tibia**) | SD of inhomogeneity of network | -17.85 | 10.38 | 2.96 | -1.11 | 0.65 | 0.33 | 0.09 |
| Biomechanical analysis (**radius**) | Stiffness, kN/mm | - | - | 1.84 | 1.80 | 1.33 | 6.08 | 0.18 |
| Biomechanical analysis (**tibia**) | Stiffness, kN/mm | - | - | 3.04 | -2.30 | 1.32 | 0.10 | 0.08 |
| | Average equivalent strain of cortical bone (C.ES) | 5463.9 | 3979.0 | 1.89 | 1.03 | 0.75 | 2.80 | 0.17 |
| | Constant | -84.79 | 58.46 | 2.10 | -0.15 | 0.57 | 0.86 | 0.15 |

**Supplementary Table S2.** Binary logistic regression model for predicting osteosarcopenia in subjects with osteoporosis/osteopenia, showing predictor variables and the β coefficients for females aged ≥ 65 (n=71). Osteosarcopenia was coded as the positive state in the analysis. Probability cut-off was determined at 0.480; *p < 0.05, ** p < 0.01.

| | | β | S.E. | Wald | Z-distribution standardization | | | |
|---|---|---|---|---|---|---|---|---|
| | | | | | β | S.E. | Odds Ratio | Sig. |
| Extended cortical analysis (tibia) | Total bone volume, $mm^3$ | - | - | 14.54 | -1.88 | 0.49 | 0.15 | 0.00** |
| Standard analysis (tibia) | Cortical thickness, mm | -5.06 | 2.01 | 6.31 | -1.13 | 0.45 | 0.03 | 0.01* |
| | Meta trabecular bone density, $mgHA/cm^3$ | -0.02 | 0.01 | 5.02 | -0.73 | 0.33 | 0.48 | 0.03* |
| | Constant | 21.75 | 6.02 | 13.06 | -0.37 | 0.3 | 0.69 | 0.21 |
